# Colorectal cancer survival among Malaysia population: data from the Malaysian National Cancer Registry

**DOI:** 10.3389/fonc.2023.1132417

**Published:** 2023-11-29

**Authors:** Nor Asiah Muhamad, Nur Hasnah Ma’amor, Izzah Athirah Rosli, Fatin Norhasny Leman, Mohd Hatta Abdul Mutalip, Huan-Keat Chan, Siti Norbayah Yusof, Nor Saleha Ibrahim Tamin, Tahir Aris, Nai Ming Lai, Muhammad Radzi Abu Hassan

**Affiliations:** ^1^ Sector for Evidence-based Healthcare, National Institutes of Health, Ministry of Health, Setia Alam, Malaysia; ^2^ Centre for Communicable Diseases Research, Institutes for Public Health, National Institute of Health, Ministry of Health, Setia Alam, Malaysia; ^3^ Clinical Research Centre, Sultanah Bahiyah Hospital, Ministry of Health, Alor Setar, Malaysia; ^4^ National Cancer Institute, Ministry of Health, Putrajaya, Malaysia; ^5^ Cancer Prevention and Control Unit, Non-communicable Disease Division, Ministry of Health, Putrajaya, Malaysia; ^6^ Institute for Medical Research, National Institutes of Health, Ministry of Health, Setia Alam, Malaysia; ^7^ School of Medicine, Taylor’s University, Subang Jaya, Malaysia; ^8^ Office of the Director General Health, Ministry of Health, Putrajaya, Malaysia

**Keywords:** colorectal neoplasm, survival time, cumulative survival, hazard, Malaysia

## Abstract

**Background:**

Colorectal cancer (CRC) is a major cause of cancer-related mortality worldwide. It is the second leading cause of cancer death in men and women in Malaysia and poses a major burden on society.

**Aims:**

To determine the overall survival rate of patients diagnosed with CRC and factors contributing to survival.

**Methods:**

Data were obtained from the Malaysia National Cancer Registry. All patients with CRC were identified, and a total of 15,515 patients were screened. A total of 5,675 CRC patients were included from January 1, 2012, to December 31, 2016. Sex, age groups, ethnic groups, stage at diagnosis, cancer sites, and status of treatment received were analysed. The Kaplan–Meier analysis was performed to estimate the 1-, 3-, and 5-year survival of CRC. The log-rank test was conducted to compare the survival between sex, age groups, ethnic groups, stage at diagnosis, cancer sites, and status of treatment received. Multiple Cox regression was conducted to determine the risk of CRC death.

**Results:**

Of 5,675, a total of 2,055 had died, 3,534 were censored, and another 86 were still alive within 5 years of CRC diagnosis. The 1-, 3-, and 5-year survival rates were 68.5%, 34.7%, and 18.4%, respectively with a median survival time of 24 months. Significant differences in survival rates of CRC were observed between age groups (*p* < 0.001), ethnic groups (*p* < 0.001), stages at diagnosis (*p* < 0.001), treatment status (*p* = 0.003), and treatment modalities (*p* < 0.001). No significant difference was observed in survival rates of CRC between sex (*p* = 0.235) and cancer sites (*p* = 0.410). Those who were 80 years old and above were found to be at higher risk of CRC death compared to those below 80 years old (adjusted hazard ratio (HR): 1.24, 95% CI 1.14–1.36). The risk of CRC death was also found four times higher among those with stage IV compared to those with stage 0 (adjusted HR: 4.28, 95% CI 3.26–5.62).

**Conclusion:**

In general, Malaysian patients with CRC had low survival rates. National health policies should focus on enhancing awareness of CRC, encouraging early screening, and developing strategies for early detection and management to reduce CRC-associated mortality.

## 1 Introduction

Cancer is one of the leading causes of death worldwide, and colorectal cancer (CRC) ranked as the second most common cancer death with 916,000 deaths reported in 2020 (1). In addition, CRC is the third most commonly diagnosed cancer in 2020, globally (2). The age-standardised incidence rate (ASR) and the age-standardised mortality rate (ASMR) for CRC were higher in men compared to women (3). In Malaysia, CRC ranks as the second most common cancer, which contributed to 13.5% of all newly diagnosed cancer cases in 2012–2016 (4). It is the most prevalent cancer in men and the second most prevalent in women (4). The ASR for men was 14.8 per 100,000, which was slightly higher than for women with 11.1 per 100,000 (4). Furthermore, the incidence of CRC was the highest among the Chinese, compared to Malays and Indians in Malaysia (4).

Colorectal cancer is characterised by uncontrolled cell growth that starts in the colon, including the ascending, transverse, descending, and sigmoid colon or rectum (5). Additionally, cancers that are located at the rectosigmoid junction also may be known as CRC, although it only contributes 10% of colorectal cancer diagnosis (6). CRC is identified as a “lifestyle or behavioural” disease and mostly attributed to non-modifiable factors such as age and gender, ethnicity, genetic predisposition, and other modifiable factors such as poor diet habits high in calories and animal fat, alcohol consumption, smoking, infection from *Helicobacter pylori*, obesity, and inactivity (7–12).

Colorectal cancer is one of the malignancies that are highly preventable and treatable with early detection (4). Early screening and detection lead to a low stage at diagnosis, in which the survival of CRC may be improved. Therefore, many public awareness and health promotion programme on CRC was conducted, and many accomplishments were seen in managing CRC, including the availability of screening tool, advancement in surgical procedure, and other treatment modalities (13). Despite that, more than 70% of CRC patients in Malaysia were diagnosed at late stages, with Malaysia having a low 5-year relative survival rate compared to other developed Asian countries (14).

Cancer survival is a crucial indicator for a better improvement in cancer management (13). By determining survival rates, one country may identify why other countries have better survival and may further implement a better strategy for the prevention, treatment, and care of a particular disease (15). Therefore, in this study, we determined the overall survival rate of CRC and factors associated with increased risks of CRC death in Malaysia based on the data from the Malaysia National Cancer Registry (MNCR) 2012–2016.

## 2 Methodology

### 2.1 Study design

A retrospective study was conducted to determine the survival rate of patients with CRC in Malaysia. This study utilised secondary data from the MNCR. This study was conducted from March to September 2022. Data were retrieved from all patients with colorectal cancer registered with MNCR from 2012 to 2016.

### 2.2 Malaysia National Cancer Registry 2012–2016

MNCR was established in 2007, and the first 5-year report for the incidence period of 2007–2011 was published in 2016 (16). The MNCR 2012–2016 is the second and latest 5-year report by the registry, which was published in 2019 (17). The registry was coordinated by the Clinical Research Centre, Sultanah Bahiyah Hospital, Ministry of Health, Alor Setar, Kedah, Malaysia. The MNCR is a national cancer database that systematically collects data on various types of cancers including colorectal, haematological, lung, breast, and cervical cancers (17). The registry includes data on cancer patients diagnosed in Malaysia between the period of January 1, 2012, and December 31, 2016. The registry captures only Malaysian citizens and residents diagnosed and treated in all the public hospitals within the Ministry of Health and other participating hospitals in Malaysia. Data were provided by appointed healthcare workers from the respective government or private health facilities across 14 states in Malaysia by a standardised cancer notification form. A total of 115,238 newly diagnosed cancer patients were registered from January 1, 2012, to December 31, 2016, of which 15,515 (13.5%) were diagnosed with colorectal cancer.

### 2.3 Data retrieval

In this study, the inclusion criteria were as follows: 1) patients diagnosed with CRC between January 1, 2012, and December 31, 2016, and 2) Malaysian citizenship. Patients were excluded when the information on the vital status of dead or alive was not available, if the patients died within a month of CRC diagnosis, or if the information on the date of CRC diagnosis was missing from the study. Therefore, a total of 5,675 patients were included in this study.

**Figure 1:**
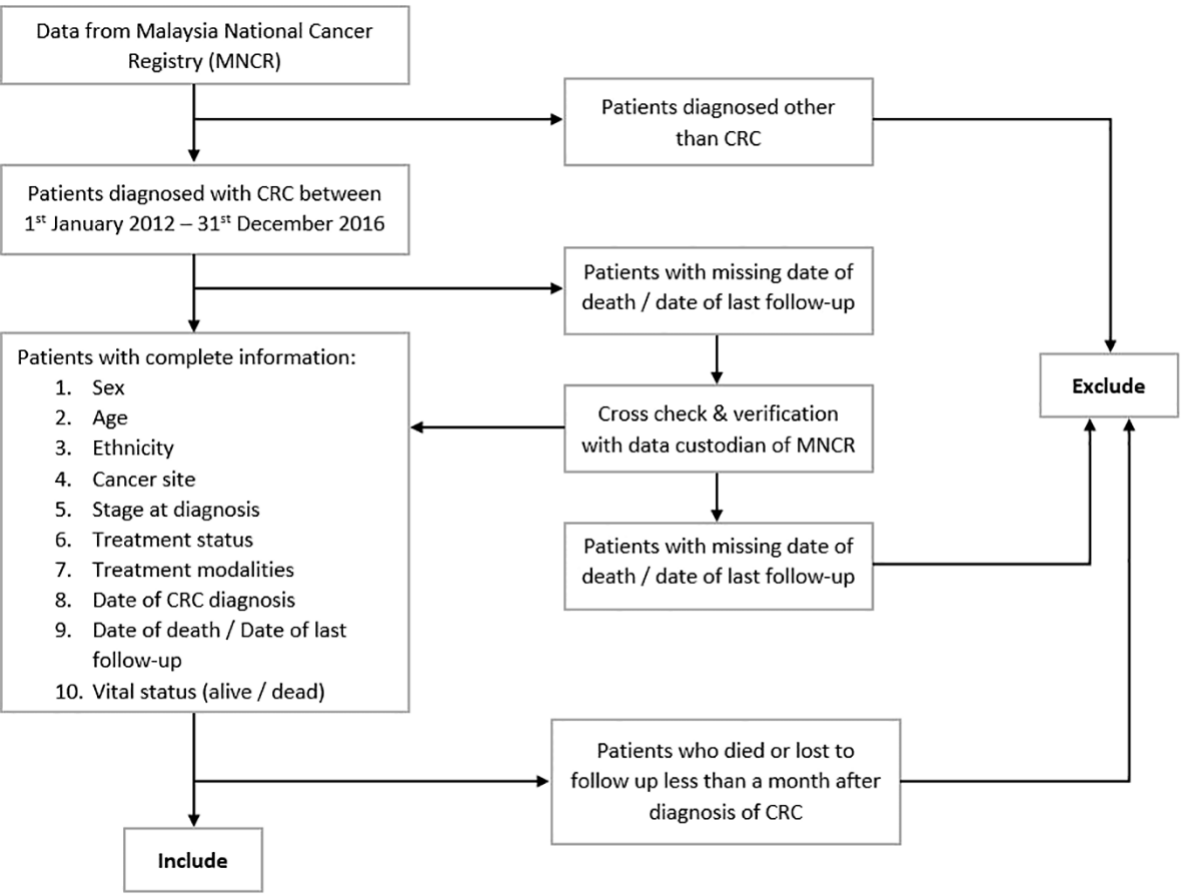
Data retrieval process of patients with CRC.

The flow diagram of the retrieval process of the patients’ data is presented in **Figure 1**. Information on sociodemographics such as sex, age, ethnicity, and clinical characteristics including cancer sites and stage at diagnosis was extracted from the records. Treatment status and treatment modalities received by the patients were also collected. The date of CRC diagnosis, the date of death, or date of last follow-up, and the vital status of the patients (alive or dead) were also retrieved. Missing information on the patient’s date of death or date of last follow-up was cross-checked and verified with the data custodian of the National Registration Department (NRD) using the patients’ National Registration Identity Card (NRIC) numbers.

### 2.4 Study variables

Sociodemographic variables included in this study were sex (male and female), age, age groups (<80 and ≥80), and ethnic groups (Malays, Chinese, Indians, and other Malaysians). Clinical characteristics included cancer sites based on the International Classification of Diseases for Oncology (ICD-O) (colon, rectosigmoid, and rectum), stage at diagnosis based on the Union for International Cancer Control/American Joint Committee on Cancer (UICC/AJCC) TNM classification system (0 to IV), treatment status (received or not), and treatment modalities received by the patients (surgery, chemotherapy, radiotherapy, other types of treatment). The categorisation of the variables was based on the category specified by the registry, except for age groups. In this study, patients’ age was divided based on the level of risk for CRC. Older adults above 80 years old with CRC had a higher risk of CRC (18).

The date of CRC diagnosis was defined by the MNCR as follows: 1) the date of first histological or cytological confirmation of the malignancy (with the exception of histology or cytology at autopsy), in which the date should be either the date when the specimen was taken (biopsy) or the date of the pathology report, 2) the date of admission to the hospital due to the malignancy, or 3) the date of first consultation at the outpatient clinic due to the malignancy. The date of death refers to the date when the patient died as stated in the death certificate or captured in the cancer registry or when cross-checked with the National Registration Department. The date of the last follow-up referred to the date on which the patient was last known to be alive by the appointed healthcare workers. If the date of death was reported, the patient’s vital status was recorded as dead; meanwhile, if the date of last follow-up was reported, the patient’s vital status was recorded as alive.

The outcome of this study was the overall survival of CRC. The event was defined as the patient’s death. Time-to-event was defined as the difference of months between the date of CRC diagnosis and the date of death or the date of last follow-up. This study evaluated patients for a period of 5 years from the date of diagnosis to death or censored. A censored patient was defined as any patient who was lost to follow-up, dropped out, or has not yet died within 5 years of CRC diagnosis. The survival was estimated to be 1 year, 3 years, and 5 years. From the data, a total of 15,515 patients with CRC were screened. Of these, a total of 5,589 patients who had incomplete information and 4,251 patients who died within a month of diagnosis were excluded. Therefore, the remaining 5,675 patients were included in the study. Out of 5,675 patients included, 2,055 had died, 3,534 were censored, and only 86 were alive within 5 years of CRC diagnosis.

### 2.5 Statistical analysis

Data were de-identified prior to the analysis by the data custodian. Data were analysed using Stata/IC statistical software version 16 (StataCorp LLC, College Station, TX, USA) and SPSS version 22.0 (IBM Corp., Armonk, NY, USA). The Kaplan–Meier method was performed to determine the 1-, 3-, and 5-year survival rates of CRC. The comparison in survival distributions between sex, age groups, ethnic groups, stage at diagnosis, cancer sites, and status of treatment received was analysed using the log-rank test. Any variables with *p-*values of less than 0.05 were further analysed using the multivariate Cox proportional hazards regression test using an automatic backward elimination method to determine the associated risk factors of CRC survival. The levels of significance were set at 0.05, and 95% confidence intervals were reported where applicable.

### 2.6 Ethical consent

We obtained ethical clearance from the Malaysia Medical Research and Ethics Committee. This study was registered under the National Medical Research Registry (NMRR-22-00326-6JB).

## 3 Results

### 3.1 Patients’ characteristics

From the study cohort, the mean age of the patients was 60.7 years (standard deviation 12.6), ranging from 13 to 96 years, of which the majority were aged less than 80 years (60.7%). Approximately 56% were men, with a mean age of 61.1 years (SD 12.3), ranging from 18 to 96 years, and 44% of the patients were women, with a mean age of 60.3 years (SD 13.0), ranging from 13 to 94 years. Among the CRC patients, 41% were Malays and 47% were Chinese, and approximately 40% suffered from stage IV, followed by stages III, II, I, and 0. Approximately half of the total patients had CRC located in the colon. In addition, approximately 82% of the patients received CRC treatment, of which most of them had surgery (35.5%); others had chemotherapy, radiotherapy, and other therapies; and some had received more than one type of cancer treatment. The characteristics of patients with CRC are presented in **Table 1**.

**Table 1:** Characteristics of patients diagnosed with colorectal cancer (n = 5,675).

Characteristics	Frequency	Percentage (%)
Sex		
Male	3,174	55.9
Female	2,501	44.1
Age groups		
<80	3,444	60.7
≥80	2,231	39.3
Ethnic groups		
Malay	2,342	41.3
Chinese	2,685	47.3
Indian	349	6.1
Others	299	5.3
Stage at diagnosis		
0	254	4.4
I	329	5.8
II	1,020	18.0
III	1,833	32.3
IV	2,239	39.5
Cancer sites		
Colon	2,930	51.6
Rectosigmoid	889	15.7
Rectum	1,856	32.7
Treatment status		
Not received	1,016	17.9
Received	4,659	82.1
Treatment modalities		
None	1,016	17.9
Surgery	2,013	35.5
Chemotherapy	540	9.5
Radiotherapy	117	2.1
Others	221	3.9
Combined	1,768	31.1

### 3.2 Survival rate of CRC

**Figure 2:**
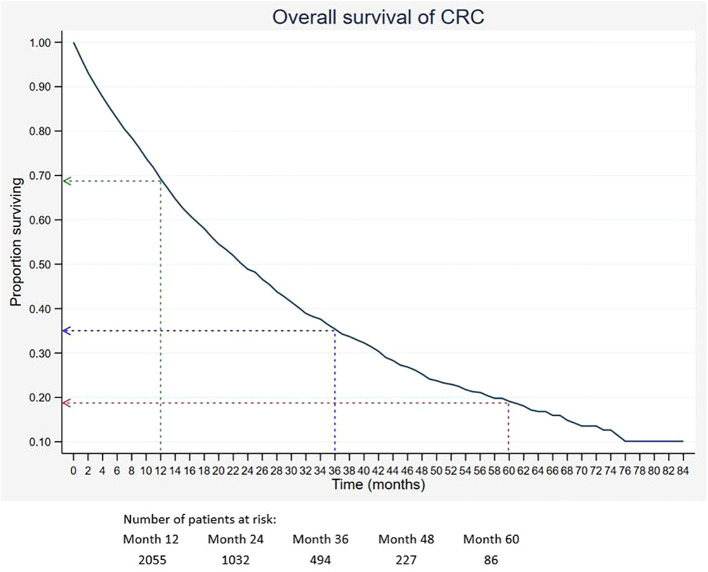
Overall survival of CRC. Green, blue and red dashed-arrows show the 1-, 3- and 5- year survival rates, respectively.

**Figure 3:**
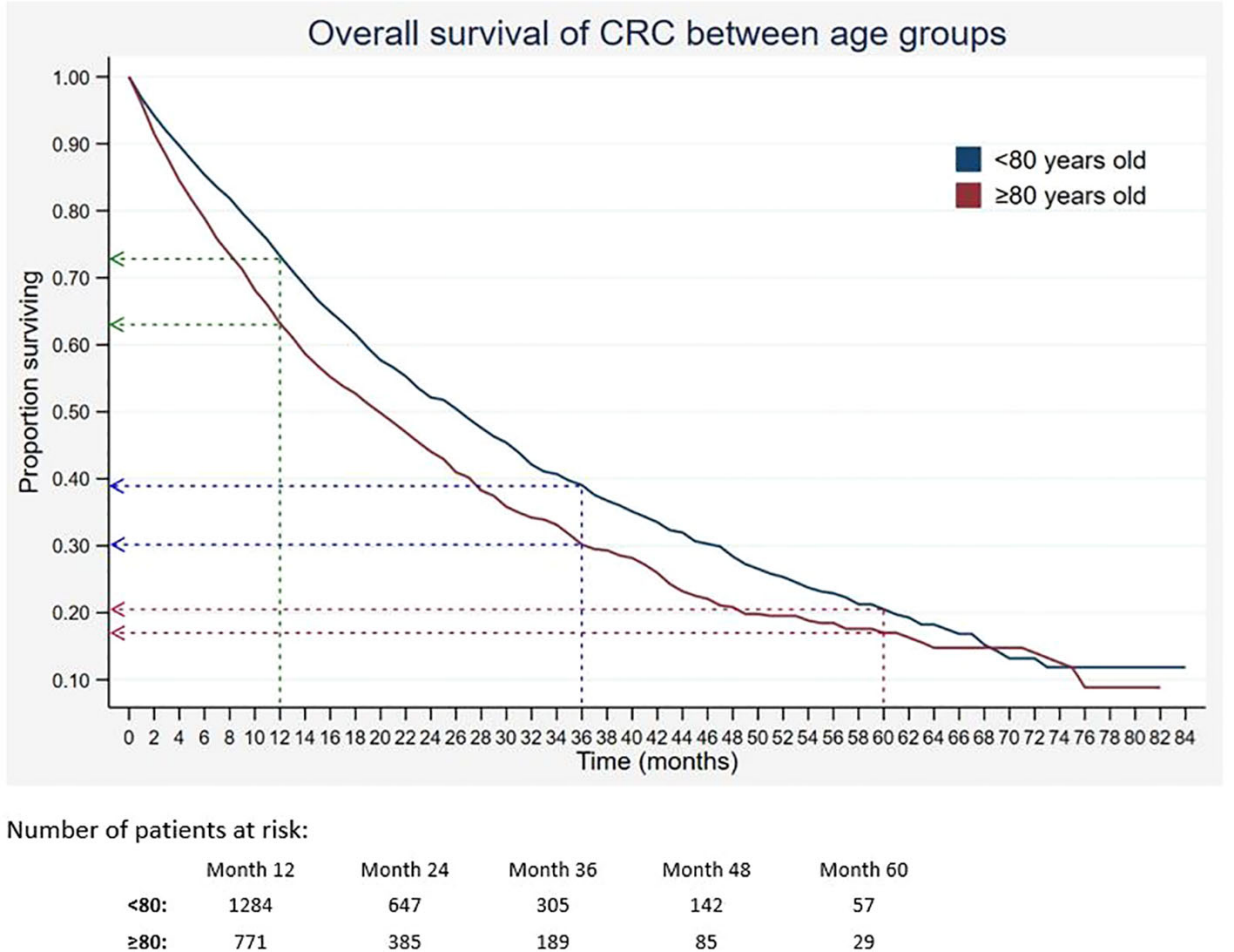
Overall survival of CRC between age groups. Green, blue and red dashed-arrows show the 1-, 3- and 5- year survival rates, respectively.

**Figure 4:**
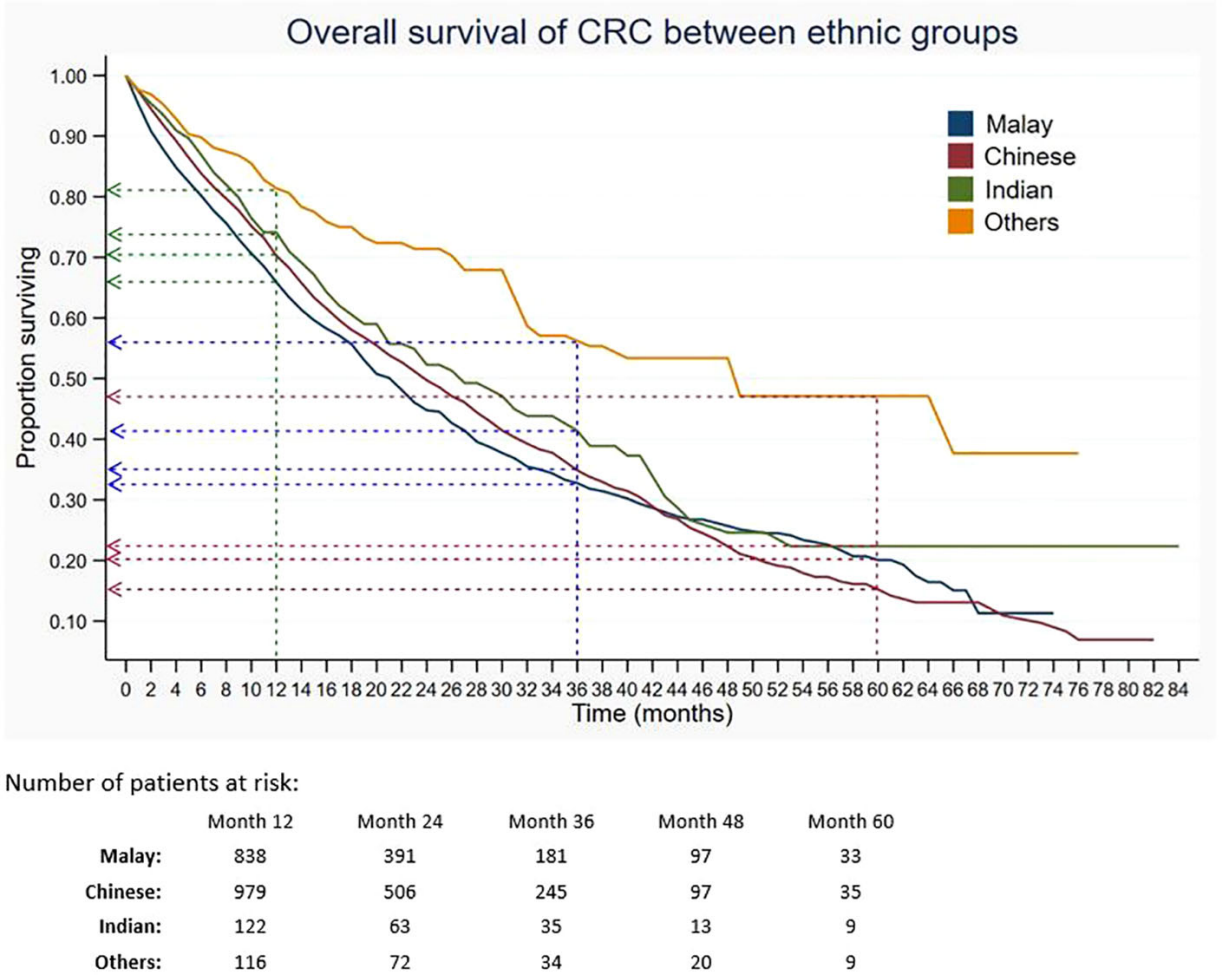
Overall survival of CRC between ethnic groups. Green, blue and red dashed-arrows show the 1-, 3- and 5- year survival rates, respectively.

**Figure 5:**
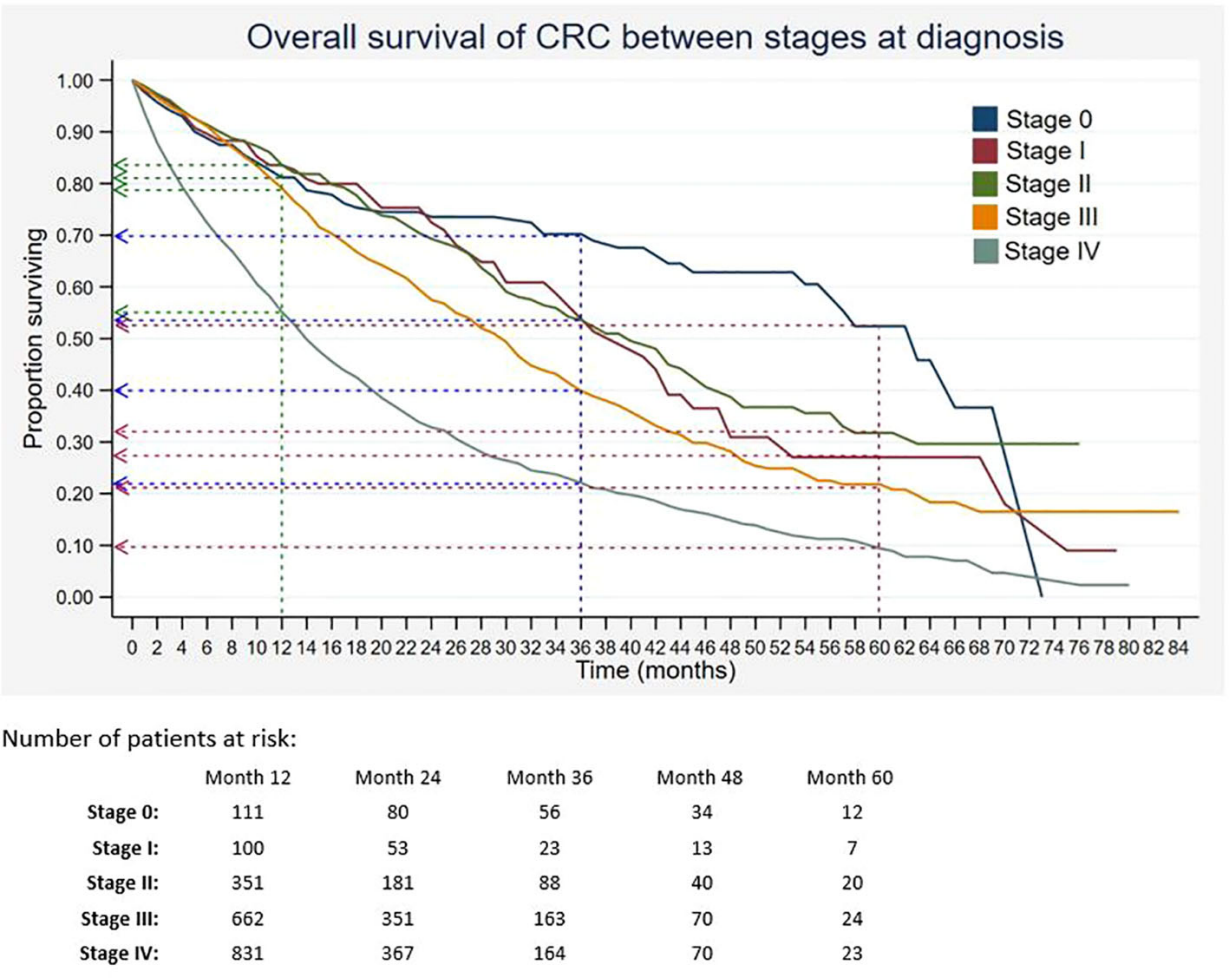
Overall survival of CRC between stages at diagnosis. Green, blue and red dashed-arrows show the 1-, 3- and 5- year survival rates, respectively.

**Figure 6:**
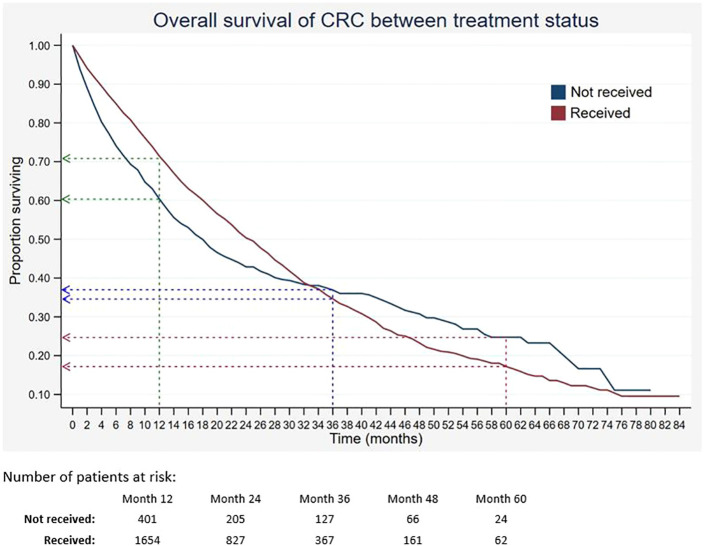
Overall survival of CRC between treatment status. Green, blue and red dashed-arrows show the 1-, 3- and 5- year survival rates, respectively.

Out of 5,675 patients included, 2,055 had died, 3,534 were censored, and only 86 were alive within 5 years of CRC diagnosis. The 1-, 3-, and 5-year overall survival rates of CRC were 68.5%, 34.7%, and 18.4%, respectively (**Table 2**). The median survival time for CRC was 24 months. The Kaplan–Meier overall survival curve of CRC is presented in **Figure 2**. From the log-rank analysis, there was no difference observed in the survival rates between male and female patients (*p* = 0.235). Similarly, no differences were seen in survival rates between cancer sites (*p* = 0.410). Conversely, there were significant differences observed in the survival rates between age groups, ethnic groups, stages at diagnosis, treatment status, and treatment modalities. The comparison of the survival curves between age groups, ethnic groups, stages at diagnosis, and treatment status is presented in **Figures 3**–**6**.

**Table 2:** One-, 3-, and 5-year survival rates and median survival time for CRC patients.

Variables	N	Survival rates (%) [95% CI]			Median survival time (months)	^a^ *p*-Value
		1-year	3-year	5-year		
**Overall**	5,675	68.5 [66.9–70.0]	34.7 [32.6–36.7]	18.4 [16.2–20.9]	24	
Sex						0.235
Male	3,174	67.5 [65.4–69.5]	34.5 [31.8–37.2]	17.2 [14.1–20.5]	23
Female	2,501	69.6 [67.3–71.9]	34.8 [31.8–38.0]	20.0 [16.5–23.6]	24
Age group						<0.001
<80	3,444	72.5 [70.6–74.4]	38.3 [35.6–41.0]	19.8 [16.7–23.2]	27
≥80	2,231	62.4 [59.8–64.9]	29.5 [26.5–32.5]	16.4 [13.3–19.9]	20
Ethnic groups						<0.001
Malays	2,342	65.1 [62.6–67.4]	32.0 [29.0–35.0]	19.3 [15.8–23.2]	22
Chinese	2,685	69.6 [67.2–71.8]	34.2 [31.2–37.1]	14.6 [11.6–18.0]	24
Indians	349	73.4 [66.8–78.9]	40.8 [32.1–49.2]	21.8 [13.0–32.0]	27
Others	299	80.8 [74.2–85.9]	56.4 [45.9–65.7]	46.3 [33.5–58.1]	49
Stages at diagnosis						<0.001
0	254	80.6 [73.6–85.9]	69.6 [60.9–76.7]	51.1 [37.9–62.9]	63
I	329	82.9 [76.2–87.9]	53.0 [40.4–64.1]	26.5 [14.2–40.5]	41
II	1,020	83.0 [79.5–86.0]	52.8 [46.6–58.6]	31.0 [23.4–38.8]	40
III	1,833	78.5 [75.8–81.0]	39.2 [35.2–43.2]	21.2 [16.7–26.0]	30
IV	2,239	54.5 [52.1–56.9]	21.6 [19.2–24.0]	9.0 [6.6–11.9]	14
Cancer sites						0.410
Colon	2,930	68.9 [66.7–71.0]	36.0 [33.2–38.8]	18.9 [15.8–22.2]	24
Rectosigmoid	889	66.0 [61.9–69.7]	34.3 [29.4–39.3]	17.4 [12.2–23.4]	23
Rectum	1,856	68.9 [66.2–71.5]	32.5 [28.9–36.1]	18.3 [14.0–23.0]	23
Treatment status						0.003
Not received	1,016	59.7 [56.1–63.1]	36.3 [32.3–40.3]	24.0 [19.3–29.0]	18
Received	4,659	70.7 [68.9–72.4]	33.9 [31.6–36.2]	16.6 [14.0–19.3]	25
Treatment modalities						<0.001
None	1,016	59.7 [56.1–63.1]	36.3 [32.3–40.3]	24.0 [19.3–29.0]	18
Surgery	2,013	67.6 [64.7–70.2]	33.7 [30.1–37.4]	16.0 [12.2–20.2]	24
Chemotherapy	540	64.4 [58.8–69.4]	27.6 [21.3–34.3]	11.3 [5.4–19.7]	19
Radiotherapy	117	62.5 [50.3–72.5]	23.4 [12.0–37.0]	16.7 [6.9–30.3]	19
Others	221	54.6 [46.3–62.2]	21.2 [13.5–30.2]	9.7 [3.9–18.9]	15
Combined	1,768	78.2 [75.6–80.5]	38.0 [34.2–41.8]	19.9 [15.7–24.5]	29

CI, confidence interval; CRC, colorectal cancer.

a Log-rank test, significant at p <0.05 (bold).

From the univariate analysis of stages at diagnosis, stratified by ethnic groups (**Table 3**), it was found that among patients with stage III and IV, there were significant differences in terms of the survival rates between different ethnic groups (both *p* < 0.001). In the stratified analysis by treatment modalities (**Table 4**), there were significant differences in the survival rates between those who suffered from stage I who received different treatment modalities (*p* = 0.007). Among patients with stage II, a significant difference in the survival rates was also observed between those who received different treatment modalities (*p* < 0.001). Similar results were found among those with stages III and IV (both *p* < 0.001).

**Table 3:** Five-year survival rate of CRC patients by stage at diagnosis, stratified by ethnic groups.

Variables	5-year survival rate (%)				^a^ *p*-Value
Stages at diagnosis	Malay n = 2,342	Chinese n = 2,685	Indian n = 349	Others n = 299	
**0**	29.9	13.8	84.2	–	0.269
**I**	18.5	0	69.5	–	0.100
**II**	22.4	30.0	22.6	45.5	0.505
**III**	13.0	13.4	10.1	62.2	**<0.001**
**IV**	6.1	2.1	–	18.4	**<0.001**

CI, confidence interval; CRC, colorectal cancer.

a Gehan–Breslow–Wilcoxon test, significant at p < 0.05 (bold).

**Table 4:** Five-year survival rate of CRC patients by stage at diagnosis, stratified by treatment modalities.

Stages at diagnosis	5-year survival rate (%)						^a^ *p*-Value
	None n = 1,016	Surgery n = 2,013	Chemotherapy n = 540	Radiotherapy n = 117	Others n = 221	Combined n = 1,768	
**0**	47.6	18.1	75.0	–	–	11.3	0.147
**I**	4.9	21.9	–	–	–	37.3	**0.007**
**II**	38.1	26.5	–	–	–	30.9	**<0.001**
**III**	14.8	15.6	13.2	–	–	19.5	**<0.001**
**IV**	8.9	2.7	–	–	11.2	4.5	**<0.001**

CRC, colorectal cancer.

**^a^** Gehan–Breslow–Wilcoxon test, significant at p < 0.05 (bold).

### 3.3 Estimates of hazards for CRC survival

Based on the result obtained from the Cox regression analysis, four factors, namely, age groups, ethnic groups, stages at diagnosis, and treatment modalities, were identified as hazards of CRC survival, after adjusting for sex and cancer sites (**Table 5**). The risk of CRC death was higher among those who were 80 years old and above (adjusted hazard ratio (HR) 1.24, 95% CI 1.14–1.36, *p* < 0.001). The Chinese had a slightly lower risk of death than the Malays (adjusted HR 0.85, 95% CI 0.78–0.93, *p* = 0.001). Patients with stage I (adjusted HR 1.51, 95% CI 1.05–2.16, *p* = 0.027), stage II (adjusted HR 1.46, 95% CI 1.08–1.97, *p* = 0.013), stage III (adjusted HR 2.17, 95% CI 1.64–2.87, *p* < 0.001), and stage IV (adjusted HR 4.28, 95% CI 3.26–5.62, *p* < 0.001) also had higher risk of CRC death compared to those with stage 0. In addition, those who received treatment had a lower risk of death compared to those who did not receive any treatment (adjusted HR 0.66, 95% CI 0.59–0.75, *p* < 0.001). Furthermore, those who received a single therapy (surgery, chemotherapy, radiotherapy, or others) had a higher risk of CRC death than those who received more than one treatment modality (all *p* < 0.001).

**Table 5:** Predictors of CRC survival (n = 5,675).

Variables	N	No. of event	^b^Adjusted HR (95% CI)	^c^ *p*-Value
Age groups				
<80 (ref)	3,444	1,139	1.00	
≥80	2,231	916	1.24 (1.14–1.36)	**<0.001**
Ethnic groups				
Malay (ref)	2,342	917	1.00	
Chinese	2,685	965	0.85 (0.78–0.93)	**0.001**
Indian	349	110	0.85 (0.69–1.03)	0.099
Others	299	63	0.45 (0.35–0.58)	**<0.001**
Stages at diagnosis				
0 (ref)	254	54	1.00	
I	329	61	1.51 (1.05–2.16)	**0.027**
II	1,020	197	1.46 (1.08–1.97)	**0.013**
III	1,833	509	2.17 (1.64–2.87)	**<0.001**
IV	2,239	1,234	4.28 (3.26–5.62)	**<0.001**
Treatment status				
Not received (ref)	1,016	468	1.00	
Received	4,659	1,587	0.66 (0.59–0.75)	**<0.001**
Treatment modalities				
Combined (ref)	1,768	563	1.00	
^a^None	1,016	468	–	**-**
Surgery	2,013	666	1.42 (1.27–1.59)	**<0.001**
Chemotherapy	540	200	1.34 (1.14–1.57)	**<0.001**
Radiotherapy	117	49	1.50 (1.12–2.00)	**0.007**
Others	221	109	1.83 (1.49–2.25)	**<0.001**

HR, hazard ratio; CI, confidence interval.

a Variable was omitted because of collinearity with variable “not received” under treatment status.

b Adjusted for sex and cancer sites.

c Multivariate Cox proportional hazards regression analysis, using backward elimination method, significant at p < 0.05 (bold).

## 4 Discussion

From this study, the 5-year colorectal cancer survival rate in Malaysia was 18.4%, which was relatively low compared to the overall survival (OS) of CRC in other countries (Thailand (44.0%), Martinique (43.8%), and Singapore (57.0%))),an countries (50.0%), the USA (58.0%), and other high-income countries (like Australia, Canada, Denmark, Norway, Sweden, and the UK), all with survival rates of approximately 67% (19–23). The calculated OS in this study was also lower as compared to the previously reported OS among CRC patients in Hospital Tuanku Ja’afar, one of the tertiary hospitals in Malaysia (46.5%) (24). The delayed presentation of CRC had contributed to the low survival in Malaysia (25). A study examining immunochemical faecal occult blood test (iFOBT) among average-risk individuals in Malaysia reported that the coverage of stool-based screening tests in Malaysia is still low (26). We found no difference in survival based on gender. This is similar to a study conducted at an oncology centre in Brazil (27). However, some studies showed that survival was lower in men (28, 29).

Survival of CRC is significantly determined by certain prognostic factors. Many studies have examined the correlation between demographic and diagnostic characteristics on the survival of patients with CRC. The current finding suggested that survival of CRC decreased with age. Almost 40% of the identified CRC patients were aged 80 years and above. Age was described as one of the predictive factors for death in cancer patients including CRC (30, 31). Furthermore, age has been linked to an increased risk of comorbid conditions among Americans, Japanese, and Danish, which lowers CRC patients’ survival rates (31–33). In light of the findings, it is important to take considerations from multidisciplinary aspects in treatment decisions, as oncological diagnosis may not be the only risk of having a low survival rate.

In this current study, ethnicity was found to be associated with CRC survival. The stage-specific analysis showed that among patients with stage IV, the Chinese had lower 5-year survival than Malays. The finding was in concordance with previous studies. Magaji et al. reported that the Chinese had lower survival of CRC compared to Malays and Indians among University of Malaya Medical Centre patients (34). Muhammad Radzi et al. also reported that the highest incidence and mortality rates were among the Chinese, causing them to have a lower CRC survival, followed by other ethnicities (35). In addition, the Chinese were reported to have less awareness of CRC than Malays, and Malays were better at identifying symptoms compared to the Chinese, contributing to delayed presentation that resulted in poorer survival (36, 37).

Other than age and ethnicity, stage at diagnosis was also found to be significantly associated with survival of CRC. Similarly, other studies showed advanced stages of CRC had lower survival as compared to early stages (19, 23, 38). In the present study, patients with stages III and IV were approximately two times and four times more at risk of dying as compared to stage 0. Previous findings from MNCR in the period between 2008 and 2009 also reported that patients diagnosed with stage IV had the highest risk of death compared to patients with earlier stages (13). In Estonia, patients with advanced stages of colon and rectal cancers have poor 5-year survival rates of 2% and 4%, respectively (38). Similarly, the relative survival at 5 years was 9% among those with late-stage diagnosis in Mumbai, India (39). It was observed that developed countries like Denmark had low 1-year survival among patients who were diagnosed with colon cancer at a late stage, approximately 41% for those with Duke’s stage D compared to 92% for stage A patients (23). This study showed that the majority of CRC cases were diagnosed in late stages. The increase in late diagnoses may be due to the ineffective screening programme in Malaysia. Additionally, there is a scarcity of national data on the incidence of adenomatous polyps or studies that evaluate the effectiveness of different CRC screening tests, which may contribute to the late diagnosis of CRC.

In addition, the 5-year survival of patients who received treatment was lower than that of those who did not receive treatment. The finding was similar to a previous study, in which the estimated 5-year survival rate of those who did not receive chemotherapy was 1.3% higher than that of those who received chemotherapy (40). On the contrary, two studies found that treatments such as systemic therapy, metastasectomy, and surgery significantly increased the relative survival for colorectal cancer (41, 42). The contradicting findings of this current study could be due to the high percentage of patients with advanced stages, in which they had poor prognostic factors that contributed to low survival despite having treatment modalities. Our results showed that patients with late stages who received surgery or chemotherapy had lower survival of CRC than those who did not receive any treatment modalities. It was stated by van den Berg et al. that standard cancer therapy was insufficient to increase survival rates of those with late stages of CRC and had poor prognostic factors (42). Therefore, the need for advancement in treatment as well as implementation of effective screening programs is important.

When compared with a previous study that analysed data of CRC patients from the MNCR record between the years 2008 and 2009, the current study that analysed patients’ records from the year 2012 to 2016 showed lower 3- and 5-year survival rates (13). The finding was due to the increased percentage of patients with advanced stages (III and IV) in the current study (72%), compared to the previous study (59%). An increased percentage of CRC patients with late stages at diagnosis was found to be one of the significant prognostic factors associated with low overall survival of the cancer (19, 20, 24). There were some studies that investigated survival among the Malaysian population (13, 24); however, the risk factors determining survival such as tumour characteristics, lifestyle, treatment, and genetic predisposition among patients with CRC were yet to be demonstrated.

This report may indicate that improvement for a better cancer control programme in Malaysia is needed. Although efforts to implement screening programs and cancer-related promotional activities have been made, the proportion of Malaysians having early screening tests is still low. The reasons why many people do not undergo screening tests are probably due to not having time, as many are busy with work, the long distance between home and hospital, and the cost of the screening test, and most of them do not feel it is necessary, as they believe they are healthy (43). Creativity in attracting people to listen and understand the importance of having an early screening test should be considered. With the undergoing development of a national population-based colorectal cancer screening programme, it is predicted that it will increase early cancer diagnosis and boost survival rates in Malaysia in the future.

### 4.1 Strengths and limitation

This study utilised data from the national registry, which has the most representative data on the incidence and mortality of CRC in different sex and ethnicities in Malaysia, in which there was at least one representative hospital across Malaysia. The hospitals were the major referral public and private hospitals treating CRC in Malaysia, providing the best data on CRC patients.

A few limitations were observed in this study. Firstly, the study had a retrospective design, which relied largely on the data extracted from the registry. Therefore, some data such as co-morbidities, family history of malignancy, and behaviours or lifestyles such as smoking and diet were unavailable. Secondly, some data on clinical characteristics including stage at diagnosis were missing and, hence, were excluded from this study. It is suggested that further studies collect more comprehensive information for a better understanding of the survival of CRC. Thirdly, considering that this study utilised a hospital-based dataset, the total of cases collected might be less than the number of cases in the population-based registration system.

## 5 Conclusion

The study reported poor CRC survival among the Malaysian population. Late presentation with advanced stage of CRC had a significant impact on the low survival of CRC. The survival rates of colorectal cancer among patients were comparable with those of some Asian countries. However, the survival rates were lower as compared to those of developed countries. Patients of Chinese ethnicity had lower survival rates compared to Malays or Indians. More advanced staging and late presentation were important predictors of colorectal cancer survival. The majority of the patients presented in an advanced stage, which caused the treatments to become ineffective. Therefore, early screening and treatment are important to improve the survival of patients with CRC. The efficiency of different screening approaches should be identified.

## Data availability statement

The datasets was obtained after receiving the ethical approval from the Malaysia Medical Research and Ethics Committee (MREC). Requests to access these datasets should be directed to the Malaysia National Cancer Registry (MNCR).

## Ethics statement

The studies involving humans were approved by Malaysia Medical Research and Ethics Committee. The studies were conducted in accordance with the local legislation and institutional requirements. Written informed consent for participation was not required from the participants or the participants’ legal guardians/next of kin in accordance with the national legislation and institutional requirements.

## Author contributions

NAM, NHM, and IAR carried out the study design, study selection, data extraction, and statistical analysis and drafted the manuscript. FL, MHAM, and CK participated in data extraction and drafted the manuscript. SY and NI participated in interoperating the data and drafted the manuscript. TA, LM, and MRAH participated in the discussion for any discrepancies and supervised the study. All authors contributed to the article and approved the submitted version.
